# CD4+ Primary T Cells Expressing HCV-Core Protein Upregulate Foxp3 and IL-10, Suppressing CD4 and CD8 T Cells

**DOI:** 10.1371/journal.pone.0085191

**Published:** 2014-01-20

**Authors:** Cecilia Fernandez-Ponce, Margarita Dominguez-Villar, Enrique Aguado, Francisco Garcia-Cozar

**Affiliations:** Department of Biomedicine, Biotechnology and Public Health (Immunology), University of Cadiz and Puerto Real University Hospital Research Unit, School of Medicine, Cadiz, Spain; New York University, United States of America

## Abstract

Adaptive T cell responses are critical for controlling HCV infection. While there is clinical evidence of a relevant role for regulatory T cells in chronic HCV-infected patients, based on their increased number and function; mechanisms underlying such a phenomena are still poorly understood. Accumulating evidence suggests that proteins from Hepatitis C virus can suppress host immune responses. We and others have shown that HCV is present in CD4+ lymphocytes from chronically infected patients and that HCV-core protein induces a state of unresponsiveness in the CD4+ tumor cell line Jurkat. Here we show that CD4+ primary T cells lentivirally transduced with HCV-core, not only acquire an anergic phenotype but also inhibit IL-2 production and proliferation of bystander CD4+ or CD8+ T cells in response to anti-CD3 plus anti-CD28 stimulation. Core-transduced CD4+ T cells show a phenotype characterized by an increased basal secretion of the regulatory cytokine IL-10, a decreased IFN-γ production upon stimulation, as well as expression of regulatory T cell markers, CTLA-4, and Foxp3. A significant induction of CD4+CD25+CD127^low^PD-1^high^TIM-3^high^ regulatory T cells with an exhausted phenotype was also observed. Moreover, CCR7 expression decreased in HCV-core expressing CD4+ T cells explaining their sequestration in inflamed tissues such as the infected liver. This work provides a new perspective on *de novo* generation of regulatory CD4+ T cells in the periphery, induced by the expression of a single viral protein.

## Introduction

Hepatitis C virus (HCV) infection is a worldwide health problem that affects more than 170 million people [1], [2] due to its tendency to develop chronic infections. Even among healthy and fully immunocompetent individuals, HCV evades clearance mechanisms, developing persistent viremia in up to 80% of infected individuals, leading to progressive hepatic fibrosis, cirrhosis and death from liver failure, as well as hepatocellular carcinoma [3]–[5].

Although mechanisms responsible for HCV persistence are not completely understood, it has been shown that failure of an adequate immune response, particularly a cellular response, underlies viral persistence [6], [7]. Studies with HCV-infected patients have revealed that during the acute phase of infection, strong and long-lasting HCV-specific CD4+ [8]–[10] and CD8+ T cell responses [11] are associated with viral clearance. But in most cases the response is insufficient for viral elimination and the virus establishes a chronic infection where CD4+ T cell responses are weak, not sustained, or even absent [12]. HCV specific CD4+ T cells have an altered proliferation rate and altered cytokine production, with a decreased IL-2 secretion [13]. HCV-specific CD8+ T cells display functional alterations, including reduced cytotoxicity and proliferative capacity and reduced secretion of antiviral cytokines, such as IFN-γ [14], [15]. There are several mechanisms that have been suggested to contribute to CD4+ T cell unresponsiveness during chronic HCV infection, among which suppression of T cell function by CD4+CD25+ T_reg_ cells is emerging as one of the most important [16]–[22]. CD4+CD25+Foxp3+ T_reg_ cells which suppress the activation, proliferation, differentiation, and effector function of many cell types, have been reported to be increased in peripheral blood, and liver infiltrates of chronically HCV infected patients [17], [23]–[25] and HCV infected hepatocytes are capable of directly inducing development of T_reg_ cells [26]. It has also been observed that HCV-specific T_reg_ cells were able to inhibit HCV-specific and non-specific CD8+ T cell proliferation and IFN-γ production ***in vitro*** [20], [27].

Accumulating evidence suggests that HCV proteins suppress host immune responses by interfering in the function of immune cells [28]–[30]. HCV is a positive-stranded RNA virus that belongs to the *Flaviviridae* family with a genome that codes for a single polyprotein of about 3000 aminoacids [31] that is cleaved by cellular and viral proteases into at least ten different mature proteins [32]. HCV-core protein lies at the N-terminal end of the immature polyprotein and forms the viral nucleocapsid. HCV-core affects several cellular processes including apoptosis and cellular transformation [33], [34], and it has also been suggested to have immunoregulatory properties [35]. HCV-core has also been shown by us and others to induce suppression when expressed in the CD4+ tumor T cell line Jurkat [21], [36], [37] the NK cell line YTS [38], or when added to CD4+ T cell cultures [39]. Doumba et al. have recently shown that addition of HCV non-enveloped particles (HCVne) to peripheral T cells induced TGF-β and IL-10, as well as expression of CTLA-4 and CD25, while CD127 expression showed a gradual decrease compatible with a regulatory phenotype with exhausted features [40].

There is evidence indicating that HCV can replicate in cells either than the hepatocyte [41], particularly in CD4+ T cell lines such as Jurkat and Molt-4 [42], being able to infect peripheral blood mononuclear cells (PBMC) *in vitro* [43]–[48] as well as lymph nodes *in vivo* [49], [50] and we and others have shown that HCV RNA is detected in peripheral blood CD4+ T cells from patients chronically infected with HCV genotype 1a [36], [42], [51]. Although the capacity of culture-produced HCV from a different genotype (2a) to infect PBMC has been challenged [52], it has also been shown that HCV from the same genotype as the one used in this manuscript (1a) [51] infects primary CD4+ T cells. HCV-Core could also enter CD4+ T cells as it has previously been suggested [23], [40], [53] and data showing that HCV-core protein is present in hepatocytes[54], non-parenchymal liver cells including lymphocytes [55] and in the serum of infected patients [56], together with data showing that it can enter hepatoma cell lines [57] further reinforces such an hypothesis. The presence of naked HCV-core particles has also been associated with active disease while lipid-associated HCV-core particles have been associated with clinical remission of liver damage [58]. It has also been reported that HCVne binding/uptake by T cells activated MAPK-p38, leading to IL-2 regulation via activation and binding of CREB, c-fos, and egr-1 transcription factors [59].

The plethora of effects taking place when analyzing infection with the whole virus as well as the scarcity and hypo-proliferative properties of T_reg_ cells *in vivo*, hamper the analysis of individual mechanisms [60]; thus, in the present work we have analyzed the effect of a single HCV protein, expressed in primary CD4+ T cells. We have shown that HCV-core expressing primary CD4+ T cells are unable to respond to CD3 plus CD28 stimulation, acquiring an anergic phenotype. Moreover, HCV-core expressing CD4+ T cells acquire suppressive functions, inhibiting T cell activation of both CD4+ and more strongly CD8+ T cells. HCV-core transduced CD4+ T cells show a phenotype characterized by an increased basal production of the regulatory cytokine IL-10 and a decreased IFN-γ production upon TCR stimulation. Core-transduced CD4+ T cells express T cell surface marker CTLA-4 and upregulate Foxp3, responsible for the suppressor ability of CD4+CD25+ T_reg_ cells [61]–[64].

The high percentage of chronicity in HCV infected patients, as well as the limited benefits of current therapies make it necessary to develop new antiviral strategies able to control HCV infection. To achieve this goal, it is desirable to know the mechanisms employed by the virus to evade the immune response and persist in the host. This work intends to identify the effects induced by HCV-core protein on CD4+ T cells and in this way our results shed light on the development of adaptive regulatory-like CD4+ T cells in the periphery, by the expression of a single viral protein.

## Materials and Methods

### Cell cultures

Human Embryonic Kidney 293FT cells (HEK 293FT™) (Life Technologies™, Carlsbad, CA, USA) were maintained in Dulbeccós modified Eaglés medium (DMEM™) supplemented with 10% (v/v) heat inactivated FBS, 10 mM Hepes, 100 U/ml penicillin and 100 µg/ml streptomycin at 37°C, 10% CO_2_.

### PBMC isolation and stimulation

Human peripheral blood samples were obtained from healthy donors upon signature of an informed consent and following approval by the Ethic sub-commission of the Puerto Real University Hospital (dependent from the Central Quality Commission), in accordance to Spanish and European Union Regulations. PBMC were isolated by density gradient centrifugation using Lymphocyte separation medium (Eurobio™, Montpellier, France). Cells were washed three times with PBS, subsequently stimulated with 1 µg/ml phytohemagglutinin-P (PHA) (Sigma™, Saint Louis, Missouri, USA) and cultured in DMEM supplemented with 1% (v/v) sodium pyruvate, non essential aminoacids, vitamins, L-arginin, L-asparragin, folic acid, 10 mM Hepes, 50 µM 2-mercaptoethanol, 100 mg/ml streptomycin, 100 U/ml penicillin (Life Technologies, Carlsbad, CA, USA) and 10% heat-inactivated FBS (Gibco) at 37°C in a 10% CO_2_ atmosphere. 40 U/ml IL-2 was added to the cultures every 48 hours, for a total of six days. Unless indicated otherwise cells were collected at day 7, washed with PBS and resuspended at 10^6^ cells/ml without IL-2.

### CD4+ and CD8+ T cell purification and stimulation

CD4+ and CD8+ T cells were immunomagnetically separated from PHA-stimulated PBMC using either Adembeads™ (Ademtech, Pessac, France), or BD IMag™ (Becton Dickinson) following manufacturer's recommendations. Purified CD4+ T cells were resuspended at 2×10^6^ cell/ml in DMEM supplemented with 10% (v/v) FBS (Gibco), 1% (v/v) NEAA, 1% (v/v) sodium pyruvate, 2 mM L-glutamine, 10 mM Hepes, 50 µM 2-mercaptoethanol, 1% (v/v) vitamins, 1% (v/v) L-arginin, L-asparragin and folic acid, 100 µg/ml streptomycin, 100 units/ml penicillin (Life Technologies™, Carlsbad, CA, USA); and cultured at 37°C in a 10% CO_2_ atmosphere. IL-2 (40 units/ml) was added every 48 hours, for a total of six days. Purity of the cultures was >99%, as assessed by FACS staining in a CyanADP-MLE™ (DakoCytomation, Beckman Coulter, Inc. Fullerton, CA, USA). At day 6 (after two transduction steps) cells were collected, washed with PBS and resuspended at 2×10^6^ cells/ml without IL-2. After a 24 hour resting period, cells were stimulated either with plate bound anti-CD3 plus soluble anti-CD28 or with the human T Cell Activation/Expansion Kit (Macs Miltenyi Biotec) or were left unstimulated to perform the experiments.

For CD8 proliferation in co-culture experiments, upon magnetic purification, CD8+ T cells were re-suspended in freezing media containing 50% supplemented DMEN, 40% FBS, 10% DMSO and stored in a Cell Freezing container (Nalgene®) at 80°C, overnight and subsequently transferred to the Liquid Nitrogen container until the day of the experiment.

### Lentiviral production

HEK 293FT™ cells (Life Technologies™, Carlsbad, CA, USA) were used as packaging cell lines to produce lentiviral supernatant. Cells were cotransfected with transfer vector pHŔSincPPT-SEWHCV-core-GFP (HCV-core) coding for the first 191 amino acids of the HCV polyprotein (serotype 1a) corresponding to HCV-core protein (or GFP as a control (pHŔSincPPT-SEWGFP) (GFP) [36]), together with plasmids pCMVΔR8.91, coding for HIV-1 GAG and POL proteins and pMD2.G for the Vesicular Stomatitis Virus G protein (VSVG). Cells were transfected in OptiMEM™ medium using Lipofectamine 2000™ (Life Technologies), according to manufactureŕs instructions. Transfection efficiency was evaluated by FACS. Supernatants were collected at 48 and 72 hours, centrifuged to remove cells and debris and concentrated using Lenti-X™ Concentrator (Clontech) to obtain a high-titer virus-containing pellet. Pellets were frozen at −80°C and stored until use. Viral titer in supernatants was determined evaluating their efficiency in infecting Jurkat cells.

### Transduction of PBMC, CD4+ or CD8+ T cells

Cells were transduced on days 2 and 4 afterPHA stimulation by spin infection (1400 g for 90 min at 32°C) at a multiplicity of infection (MOI) of 10 in the presence of IL-2 (40 units/ml) and Polybrene (4 µg/ml) (Sigma, St. Louis, MO). GFP expression was monitored at several time points by FACS, being in a range of 35–90% at the time of the assay (>85% for the co-culture assays, 35–90% for the rest of experiments). Cells were cultured for 48 additional hours, after the last transduction, before performing the experiments. Subsequently, cells were left resting for 24 hours and stimulated using loaded anti-biotin MACSiBead Particles (bead-to-cell ratio 1∶1). Cells were cultured at a density of 2×10^6^ cells per mL at 37°C and 5% CO_2_ atmosphere, for 24 additional hours.

### Cell surface receptor staining

Cell surface receptors were stained with the following antibodies: APC-Cy7- labeled mouse anti-human CD25 (BD Biosciences, San Diego, CA, USA), PE-Cy7- labeled mouse anti-human CD8 (BD Biosciences), biotin-conjugated mouse anti-human CD4 (BD Biosciences), followed by streptavidin-PE (BD Biosciences), APC-labeled mouse anti-human CTLA-4 (BD Biosciences), APC-labeled rat monoclonal anti-human TIM-3 (R&D Systems™), APC-labeled mouse anti-human CD279 (PD-1) (eBioscience®), APC-labeled mouse anti-human CD95 (Fas/APO-1), Alexa Fluor® 647-labeled mouse anti-human CD194 (CCR4) (Biolegend™), Alexa Fluor® 647-labeled mouse anti-human CD196 (CCR6) (Biolegend™), APC-labeled mouse anti-human CD197 (CCR7) (Biolegend™), Alexa Fluor® 647-labeled mouse anti-human CD183 (CXCR3) (Biolegend™), APC-Cy7-labeled mouse anti-human CD62L (Biolegend™), eFluor® 660-labeled mouse anti-human CD127 (eBioscience®), Alexa Fluor® 647-labeled mouse IgG1 κ Isotype control (BD™ Phosflow), Pacific Blue™-labeled mouse IgG2a, κ Isotype Control (BD Pharmingen™) and V450-labeled mouse IgG2b, κ Isotype Control (BD Horizon™).

### Intracellular staining

CD4+ or CD8+ T cells were collected at day six (after transfections at days 2 and 4) and rested without IL-2 for 24 hours at 10^6^ cells/ml. Subsequently, cells were stimulated by means of the T Cell Activation/Expansion Kit human (Macs Miltenyi Biotec®) using a cell bead ratio of 1∶1, or left unstimulated. GolgiStop™ (BD Biosciences) was added one hour after stimulation. Six hours after stimulation, cells were collected labeled with corresponding surface antibodies and subsequently incubated in the Cytofix/Cytoperm™ kit (BD Biosciences) prior to intracellular staining with APC-labeled mouse anti-human Foxp3 (eBioscience), APC-mouse anti-human IFN-γ, PE-labeled mouse anti-human IL-2, APC-rat anti-human IL-10, APC-rat anti-human IL-2 (all from BD Biosciences) or APC-mouse anti-human TGF-β1 (Lifespan Biosciences). Briefly, cells were washed with PBS and fixed using mild conditions (in 2% PFA, pH 7.4 for 15 minutes at 4°C), to preserve GFP expression. Cells were subsequently washed twice with Perm/Wash buffer and left in the same buffer for 20 minutes at room temperature to be permeabilized. Antibodies were added and incubated for 1 hour at 4°C. After two washes with PBS, intracellular staining was monitored by flow cytometry.

### Foxp3 and IL-10 double staining

For Foxp3 and IL-10 double staining, untransduced, GFP or HCV-core expressing CD4+ T cells were washed, counted and cultured for 24 hours without IL-2 in a density of 2×10^6^ cells/ml. Subsequently, each sample was divided in two wells (stimulated vs. unstimulated controls). CD4+ T cells were stimulated with the loaded Anti-biotin MACSiBead Particle (bead-to-cell ratio 1∶1) using the T Cell Activation/Expansion Kit human (Macs Miltenyi Biotec™).

After 5-hour incubation, cells were washed with PBS 0.5% FBS 2 mM EDTA to perform the IL-10 and Foxp3 staining. For IL-10 the human IL-10 secretion assay detection kit (APC) (Macs Miltenyi Biotec ™) was used. Dead cells were excluded by means of the Live/Dead® fixable dead cell stain kit (Life Technologies®). Cells were fixed and permeabilized by means of the Foxp3 fixation/permeabilization kit (eBioscience™) and Foxp3 was stained with V450 mouse anti-human Foxp3 (BD Bioscience Horizon™) according to manufacturer's instructions. Briefly, cells were washed in 1.5 mL cold buffer, centrifuged, resuspended in 90 µL cold media and 10 µL IL-10 catch reagent and incubated for 5 minutes on ice. Warm (37°C) medium (1 ml) was added to dilute cells according to the expected number of IL10 secreting cells. Cells were incubated for 45 min. at 37°C under slow continuous rotation washed and resuspended in 90 µL cold buffer. Upon 10 min incubation with IL-10 detection antibody (APC) (10 µL) in the presence of Live/Dead® Fixable dead cell stain kit (Life Technologies™) cells were washed and subsequently fixed and permeabilized for Foxp3 staining. Staining was monitored in a CyanADP-MLE™ (DakoCytomation, Beckman Coulter, Inc. Fullerton, CA, USA).

### Co-culture assays for intracellular staining

Untransduced CD8+ T cells (10^6^ cells/ml in 1 ml) were added to 0.25×10^6^ or 0.5×10^6^ untransduced, GFP transduced or HCV-core transduced CD4+ T cells (0.25∶1 and 0.5∶1 transduced:responder ratio) and stimulated with anti-human CD3 (1 µg/ml) and anti-human CD28 (1 µg/ml). For intracellular staining, GolgiStop™ was added to inhibit protein secretion an hour after stimulation and cells were incubated for a total of six hours before performing cytokine labeling.

### CD4+/CD8+ T cell co-culture proliferation assay

Transduced CD4+ T cells were irradiated (3000 rad) using a X-Ray irradiator Cabinet and added at a 1∶6 transduced:responder ratio to untransduced CD8+ T cells (2×10^6^ cells/ml) from the same donor (kept frozen while CD4+ T cells were blasted and transduced). Cells were stimulated with loaded antibiotin MACSiBead Particles (human T-cell activation/expansion kit; Macs Miltenyi Biotec) at a 1∶1 bead-to-cell ratio and 5 µM EdU pulses were added at times 0 h, 24 h, 48 h and 72 h (a non EdU treated control was included as a negative staining control). At 96 h, cells were collected, stained with anti-CD8, and proliferation analyzed using Click-iT™ EdU Flow Cytometry Assay Kits (Life Technologies™) [65], following manufacturer recommendation.

### Statistical analysis

Student's two-sample T test was used, assuming unequal variances when comparing replicates from the different conditions with the corresponding controls within each experiment.

## Results

### Expression of HCV-core inhibits IL-2 production by PBMC from healthy donors

**Figure 1:**
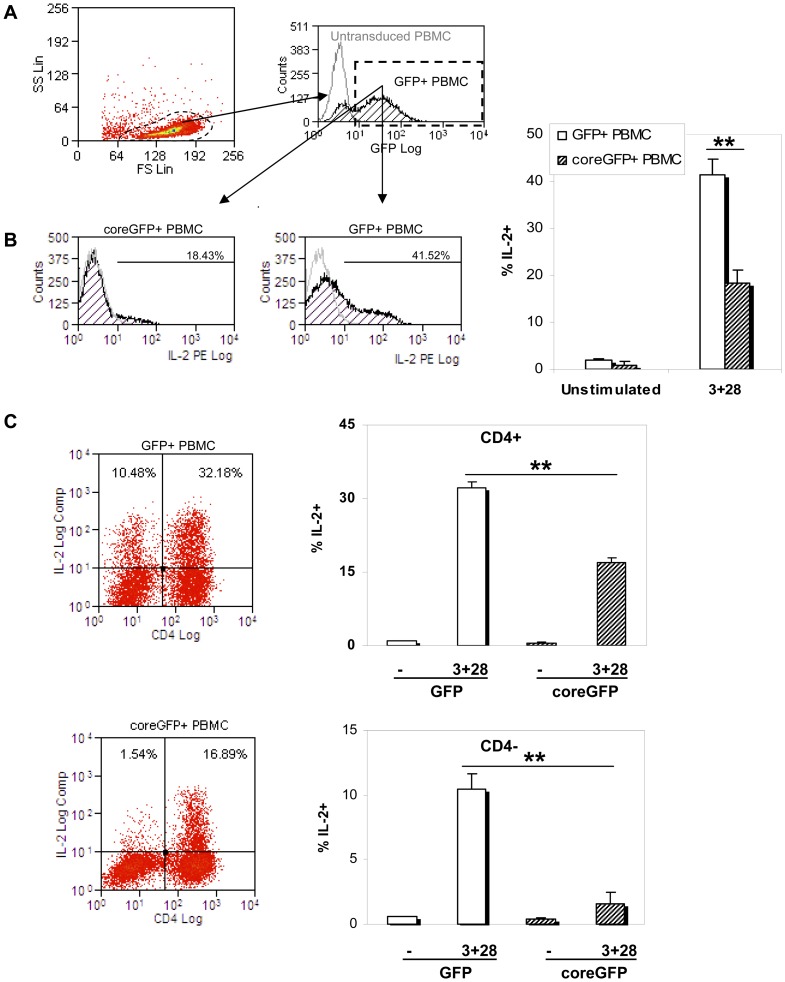
IL-2 production by unfractionated PBMC expressing HCV-core. PBMC transduced with a lentiviral vector coding for HCV-core protein fused to GFP or GFP alone as a control, were rested for 24 hours without IL-2 after six days in culture and stimulated with anti-CD3 plus anti-CD28. Intracellular IL-2 staining was analyzed by FACS. **A**. Gating strategy: GFP expressing lymphocytes were gated according to their FSC and SSC as well as green fluorescent features. **B**. IL-2 production by HCV-core expressing PBMC. Histograms are a representative experiment out of four performed with a different donor each. GFP or HCV-core transduced PHA-blasts (black striped curves). Grey open curves correspond to isotype control staining. Bar diagram represents mean±SD of the % of PBMC that produce IL-2 from four independent experiments. **C**. IL-2 production of CD4+ or CD4- cells in unfractionated PBMC. PBMC were processed as described above and stained with anti-CD4 before IL-2 staining. Dot plots show CD4 staining versus IL-2 production of electronically gated GFP (or HCV-core). Bar diagrams show mean±SD of the percentage of IL-2-producing cells for each subpopulations from four independent experiments (**p<0.01).

As it has been previously shown by others and us in the tumor T cell line Jurkat that expression of HCV-core blocks IL-2 production, we aimed to test the effect of HCV-core in primary, non-tumor T cells. PBMC were isolated from healthy donors, stimulated with PHA and transduced with lentiviral vectors coding for HCV-core or GFP as a control. Experiments with untransduced cells were run in parallel to rule out an effect due to transduction. As it has been reported that naïve cells are resistant to anergy induction *in vitro* [66]–[68], cells were stimulated with PHA and cultured for six days prior to the experiment. PHA blasts were subsequently stimulated with anti-CD3 plus anti-CD28 and the frequency of IL-2 producing cells was determined by intracellular cytokine staining. As shown in figure 1, IL-2 production by stimulated PBMC expressing HCV-core was decreased when compared to GFP-expressing PBMC (18.43±2.71% IL-2+ cells versus 41.42±3.12%, respectively). There were no significant differences in the percentage of IL-2 producing cells between untransduced and GFP-expressing PBMC (data not shown).

As previous results in T cells were obtained from CD4+ T cells lines, transduced PBMC were stained for CD4 expression to analyze HCV-core effect in CD4+ and non CD4 T cells. As shown in figure 1C, HCV-core expressing CD4+ T cells showed a decrease in IL-2 production (Fig. 1C, 32.18±1.12% IL-2+ GFP expressing CD4+ T cells compared to 16.89±0.97% IL-2+ HCV-core-expressing CD4+ T cells), but the most significant decrease in IL-2 production occurred in non CD4 T cells, where there was up to a 7-fold decrease (10.48±1.12% in GFP-expressing CD4- T cells compared to 1.54±0.97% in HCV-core expressing CD4- T cells). Thus our data show that HCV-core expression in PHA-activated PBMC results in a decrease in IL-2 production by CD4+, suggesting an even more profound effect in non CD4+ T cells.

### HCV-core expression inhibits IL-2 production of CD4+ but not CD8+ T cells

**Figure 2:**
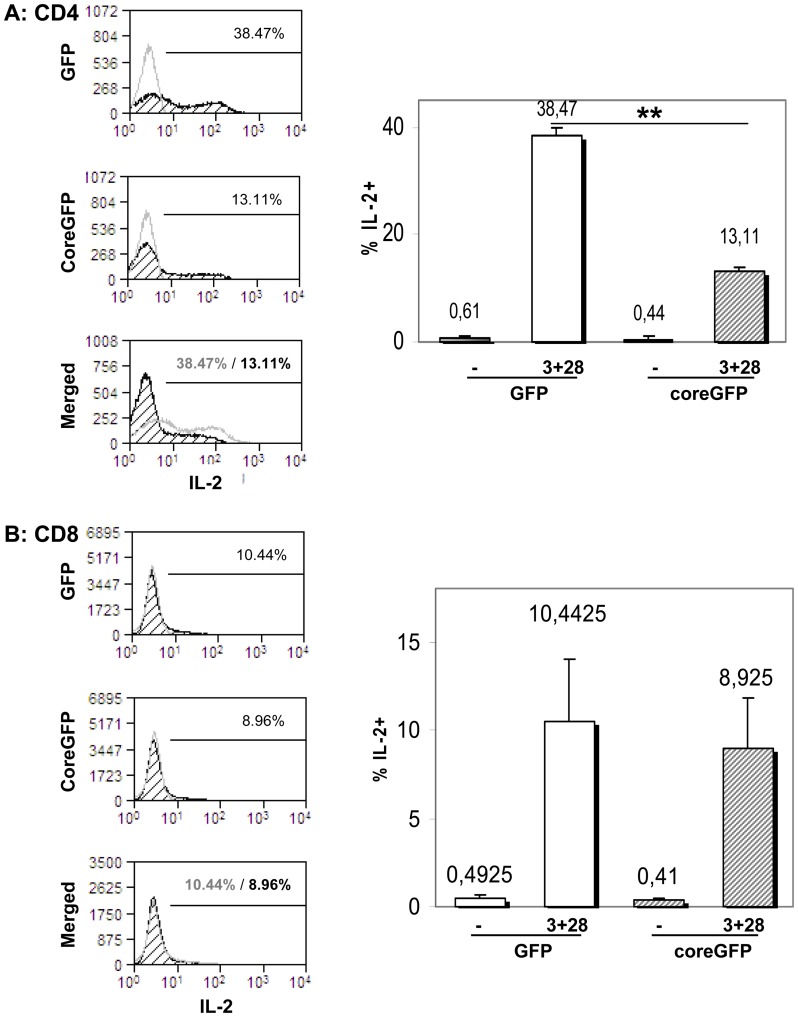
HCV-core protein inhibits IL-2 production by purified CD4+ but not CD8+ T cells. Purified CD4+ or CD8+ T cells expressing HCV-core protein or GFP as a control were rested for 24 hours and subsequently stimulated with anti-CD3 plus anti-CD28. Four hours later IL-2 production was measured by intracellular staining with an APC-labeled anti-IL-2 antibody. **A** Left panel: Histograms showing stimulation induced IL-2 production of CD4+ T cells transduced with GFP (upper histogram), HCV-core (middle) or merged (bottom). Right panel: Bar diagram showing mean±SD of four different experiments. **B**. Same as in A but for CD8 transduced cells. (**p<0.01).

To analyze whether HCV-core has a direct effect in both T cell subpopulations we analyzed separately the effect of HCV-core expression on IL-2 production by CD4+ or CD8+ T cells. Thus, CD4+ and CD8+ T cells were purified 24 hours after addition of PHA and transduced with HCV-core or GFP as a control. After six days in culture, cells were stimulated with anti-CD3 plus anti-CD28 and stained for intracellular IL-2 production (Fig. 2). Purified CD4+ T cells transduced with HCV-core showed a decrease in IL-2 production similar to that observed in the corresponding subpopulations when unfractionated PBMC cultures were analyzed, with 38.47±1.45% of IL-2+ GFP-expressing CD4+ T cells compared to 13.11±0.76% in HCV-core transduced CD4+ T cells (Fig. 2A). On the contrary there were no significant differences between GFP and HCV-core transduced CD8+ T cells (Fig. 2B), in clear contrast to the data obtained in PBMC (10.44±3.54% in GFP expressing CD8+ T cells versus 8.93±2.91% in HCV-core transduced CD8+ T cells). Untransduced CD8+ T cells showed the same behavior as GFP transduced control cells (data not shown).

### Core-expressing CD4+ T cells inhibit CD4+ and CD8+ T cell IL-2 production

**Figure 3:**
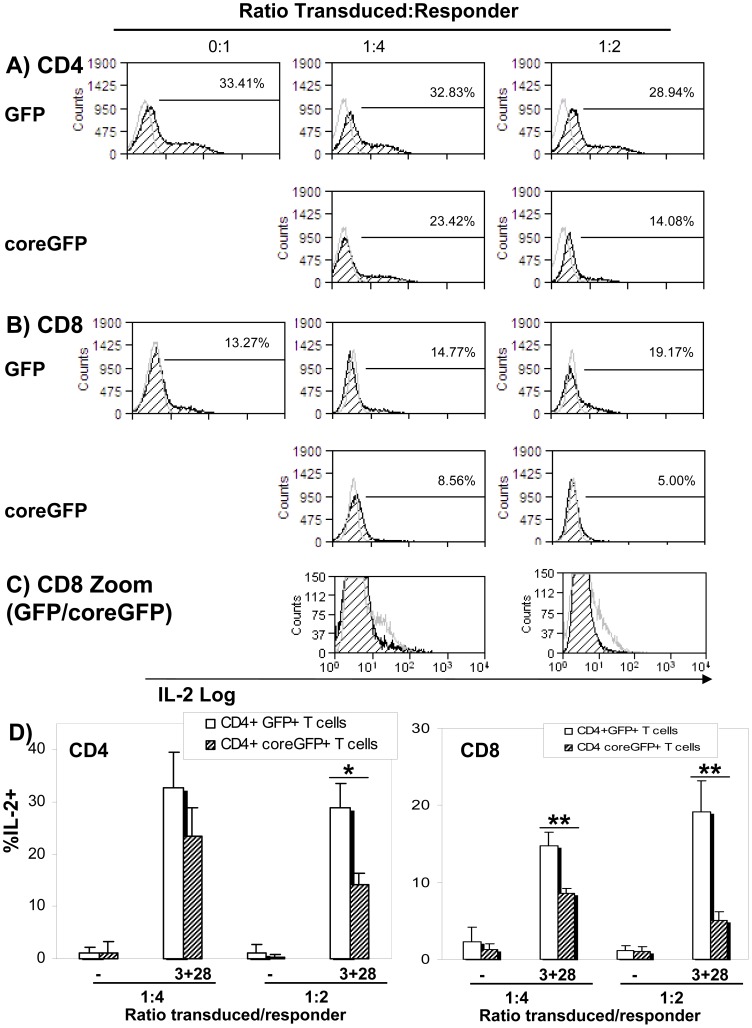
Core-expressing CD4+ T cells inhibit IL-2 production by either CD4+ or CD8+ T cells. Purified CD4+ or CD8+ T cells were co-cultured with CD4+ T cells either un-transduced or transduced with HCV-core or GFP, at two CD4+:responder ratios (1∶4 and 1∶2) and subsequently stimulated with anti-CD3 plus anti-CD28. Two hours later GolgiStop™ was added to the cultures and the cells were incubated for five more hours before IL-2 staining. CD8 surface staining was performed prior to fixation. Untransduced responder cells were identified by their GFP negativity (CD8 staining was additionally used for the CD8 experiments). Histograms represent frequency of IL-2 production of untransduced CD4+ T cells (**A**) or CD8+ T cells (**B**) in contact with GFP-transduced CD4+ T cells (upper row) or with HCV-core expressing CD4+ T cells (lower row). Grey open curve corresponds to isotype control. **C**) Zoom of the overlapped histograms from B corresponding to the frequency of untransduced CD8+ T cells producing IL2 when in contact with GFP transduced CD4+ T cells (grey curve) or with HCV-core expressing CD4+ T cells (black striped curve). **D**). Bar diagram corresponding to mean±SD of five independent experiments from different donors of the percentage of IL-2 producing CD4+ (left) or CD8+ (right) T cells in the presence of HCV-core expressing CD4+ T cells or GFP expressing CD4+ T cells. (*p<0.05, **p<0.01).

As CD8+ T cells were not directly affected by HCV-core expression, we wanted to analyze whether there was a regulatory effect mediated by HCV-core expressing CD4+ T cells. Thus, HCV-core or GFP-transduced CD4+ T cells were added to CD4+ or CD8+ untransduced “responder” cells at ratios 1∶2 or 1∶4 (transduced:responder ratio). Cells were purified from PHA-activated PBMC 24 hours after stimulation and cultured for six days as described above. A proportion of these cells were cultured separately and transduced with HCV-core or GFP. The percentage of transduction in this cultures was >85% in all the experiments. Transduced and responder cells were cultured together and stimulated with anti-CD3 plus anti-CD28. IL-2 production was measured in un-transduced responder cells (GFP negative) 6 hours after stimulation by intracellular cytokine staining (Fig. 3). Figure 3A shows IL-2 production by responder CD4+ T cells co-cultured with HCV-core expressing CD4+ T cells or GFP expressing CD4+ T cells for transduced:responder ratios 1∶2 and 1∶4 (black striped curves); curves in grey correspond to unstimulated controls. HCV-core inhibited IL-2 production by responder CD4+ T cells at a ratio of 1∶2 when compared to responders cultured in the presence of GFP transduced cells (or untransduced cells –data not shown-) (28.94±4.67% in CD4+ T cells cultured with GFP expressing CD4+ T cells compared to 14.28±2.25% in CD4+ T cells in contact with HCV-core expressing CD4+ T cells) (Fig. 3A and D).

Inhibition mediated by HCV-core transduced cells was even more pronounced when the effect on CD8+ T cell was analyzed (Fig. 3B, C and D). HCV-core expressing CD4+ T cells inhibited IL-2 production by CD8+ T cell at both ratios tested (Fig. 3B, C and D). At ratio 1∶4, the percentage of IL-2 producing CD8+ T cells was 14.77±1.69% when co-cultured with GFP expressing CD4+ T cells and 8.56±0.68% when co-cultured with HCV-core expressing CD4+ T cells. The inhibition was higher at a 1∶2 ratio, with 19.17±4.06% of CD8+ T cells producing IL-2 when co-cultured with GFP expressing CD4+ T cells compared to 4.96±1.21% of IL-2 producing CD8+ T cells in the presence of HCV-core expressing CD4+ T cells.

### Core-expressing CD4+ T cells inhibit CD8+ T cell proliferation upon anti-CD3 plus anti-CD28 stimulation

**Figure 4:**
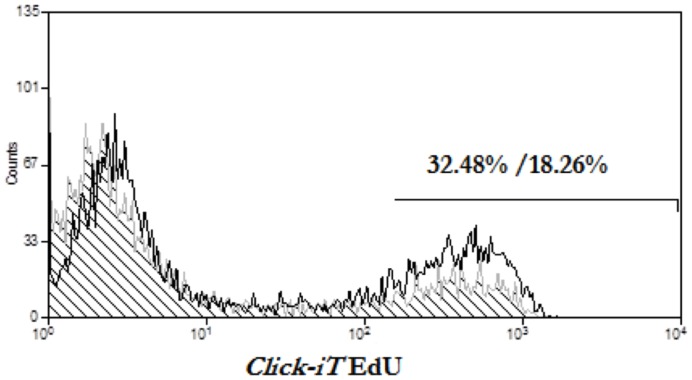
Core-expressing CD4+ T cells inhibit CD8+ T cell proliferation upon anti-CD3 plus anti-CD28 stimulation. HCV-core transduced or GFP-transduced CD4+ T cells were co-cultured with untransduced purified CD8+ T cells from the same donor at 1∶6 transduced CD4+: responder CD8+ ratio, stimulated with anti-CD3 plus anti-CD28 and cultured in the presence of EdU. EdU incorporation, was analyzed by FACS 96 hours after stimulation. Histograms represent EdU staining from untransduced CD8+ T cells in the presence of HCV-core expressing CD4+ T cells (black striped curve) or GFP expressing CD4+ T cells (grey open curve). A representative experiment out of three independent experiments from three independent donors is shown (*p<0.05)

We next wanted to analyze whether the decrease on IL-2 production by CD8+ T cells cultured in the presence of HCV-core expressing CD4+ T cells was accompanied by a decrease in CD8+ T cell proliferation. Untransduced, GFP transduced or HCV-core GFP transduced CD4+ T cells were co-cultured with purified responder CD8+ T cells from the same donor at 1∶6 transduced CD4+ : responder CD8+ ratio, and stimulated with anti-CD3 plus anti-CD28. EdU incorporation was measured at 96 hours by FACS (Fig. 4). CD8+ T cells alone were also included in the experiments as a control (data not shown). 32.48% of CD8+ T cells in contact with GFP expressing control CD4+ T cells had divided, compared to 18.26% of CD8+ T cells co-cultured with HCV-core transduced CD4+ T cells, which represents almost a 2-fold decrease in cell division. Representative data out of three independent experiments from three independent donors is shown (Fig. 4).

### Increased expression of CTLA-4 and Foxp3 in HCV-core expressing CD4+ T cells

As HCV-core expressing CD4+ T cells showed a “regulatory-like” function, we were interested in analyzing some features associated with a regulatory phenotype. Expression of CD25 and GITR was not significantly increased in resting HCV-core transduced cells and CD25 showed only a slight increase upon stimulation (data not shown).

**Figure 5:**
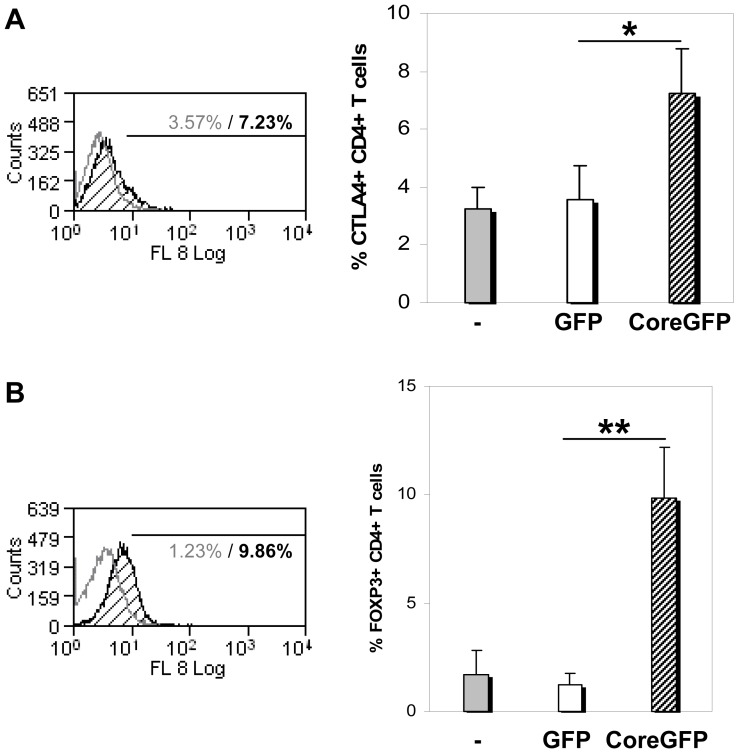
CTLA-4 and Foxp3 expression in unstimulated HCV-core expressing CD4+ T cells. Purified CD4+ T cells were transduced with lentiviral vector coding for HCV-core or GFP as a control. **A. CTLA-4 expression**. Resting HCV-core or GFP transduced CD4+ T cells were stained for CTLA-4 surface expression. Histogram represents CTLA-4 intensity in HCV-core expressing cells (black striped curve) compared to GFP expressing CD4+ T cells (grey open curve). Numbers indicate percentage of CTLA-4 positive cells based on a negative isotype control. Bar diagram (right) corresponds to mean±SD of the percentage of CTLA-4 positive cells for each sample, including untransduced CD4+ T cells (grey bar) of four independent experiments from different donors. **B. Foxp3 expression**. Histogram represents the percentage of Foxp3+ cells for HCV-core (black striped curve) or GFP expressing CD4+ T cells (grey curve). Bar diagram corresponds to mean±SD of the percentage of Foxp3+ CD4+ T cells from four independent experiments from different donors. (*p<0.05, **p<0.01).

CTLA-4 staining showed a slight increase in resting HCV-core expressing CD4+ T cells compared to control cells (3.57±1.2% CTLA4+ cells in GFP expressing CD4+ versus 7.23±1.6% in HCV-core expressing CD4+ T cells) (Fig. 5A), while Foxp3 expression showed the most robust increase (9.86±2.31% Foxp3+ cells in HCV-core expressing CD4+ T cells versus 1.23±0.54% in GFP expressing CD4+ T cells) (Fig. 5B). Foxp3 expression was maintained from day 5 to day 10 post-transduction, the last day tested (data not shown).

### Increased IL-10 and decreased IFN-γ secretion in HCV-core expressing CD4+ T cells

**Figure 6:**
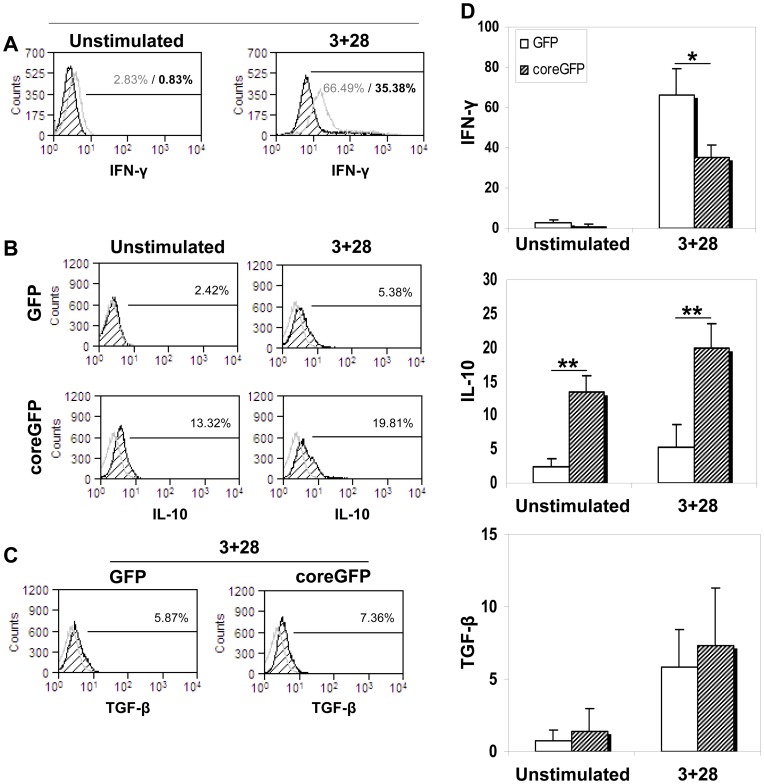
IFN-γ, IL-10 and TGF-β production in HCV-core expressing CD4+ T cells. CD4+ T cells were transduced with lentiviral vectors coding for HCV-core or GFP as a control. GolgiStop™ was added to the cultures one hour after stimulation and 5 hours later cells were collected and stained for intracellular cytokine expression. **A**. **IFN-γ is decreased in HCV-core expressing CD4+ T cells upon stimulation**. Histograms represent IFN-γ expression in HCV-core (black striped curve) or GFP (grey curve) expressing CD4+ T cells, before (left histogram) and after (right) stimulation. Percentage was calculated based on a negative isotype control. **B. IL-10 expression**. Histograms represent IL-10 intensity in GFP (upper row) or HCV-core (lower row) expressing CD4+ T cells before (left column) and after stimulation with anti-CD3 plus anti-CD28 (right column). Grey curves correspond to the negative isotype control. **C. TGF-β expression.** Histograms represent TGF-β expression in HCV-core (right histogram) or GFP (left histogram) expressing CD4+ T cells after stimulation. **D**. Bar diagrams represent mean±SD of the percentage of positive staining for each cytokine out of three independent experiments each from independent donors. (*p<0.05, **p<0.01).

We next wanted to analyze whether cytokines other than IL-2 were also affected in HCV-core transduced cells. HCV-core expressing CD4+ T cells, both stimulated and resting, showed an increase in IL-10 secretion, while the decrease in IFN-γ was only apparent upon CD3 plus CD28 stimulation (Fig. 6). TGF-β was not significantly affected by HCV-core expression. These results suggest that HCV-core expression in CD4+ T cells induces, a phenotype characterized by an increase in IL-10, CTLA-4 and Foxp3 and a decrease in IFN-γ and IL-2.

### Co-expression of IL-10 and Foxp3 in HCV-core expressing CD4+ T cells

**Figure 7:**
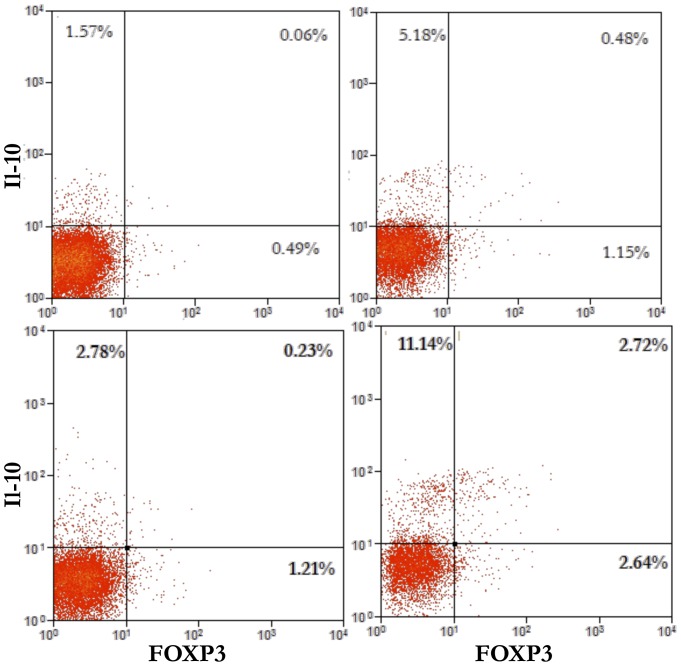
Co-expression of IL-10 and Foxp3 in HCV-core expressing CD4+ T cells. HCV-core or GFP-transduced CD4+ T cells were co-stained for IL-10 and Foxp3. Dot plots show staining for IL-10 and Foxp3 after positive gating of transduced cells and exclusion of dead cells, for unstimulated (upper panels) or stimulated (lower panels) GFP (left panels) or HCV-core (right panels) transduced CD4+ T cells. Numbers in quadrants represent percentage of positive cells in each quadrant.

As we have shown that HCV-core expressing CD4+ T cells express both Foxp3 and IL-10 inhibitory markers, we were interested in analyzing whether those two markers were co-expressed in the same cell. In unstimulated cells, although there is an increase in IL-10 secretion in HCV-core expressing cells, only a portion 0.48% of the 5.66% cells that secrete IL-10, co-express Foxp3 (Fig. 7 upper panels). Anti-CD3 plus anti-CD28 stimulation (Fig. 7 lower panels) induces a considerable increase in the percentage of IL-10 secreting cells mostly in HCV-core transduced CD4+ T cells, however, only a portion (2.72%) of those (13,86%) IL-10 positive cells, co-express Foxp3.

### Increased PD-1 in resting HCV-core expressing CD4+ T cells

**Figure 8:**
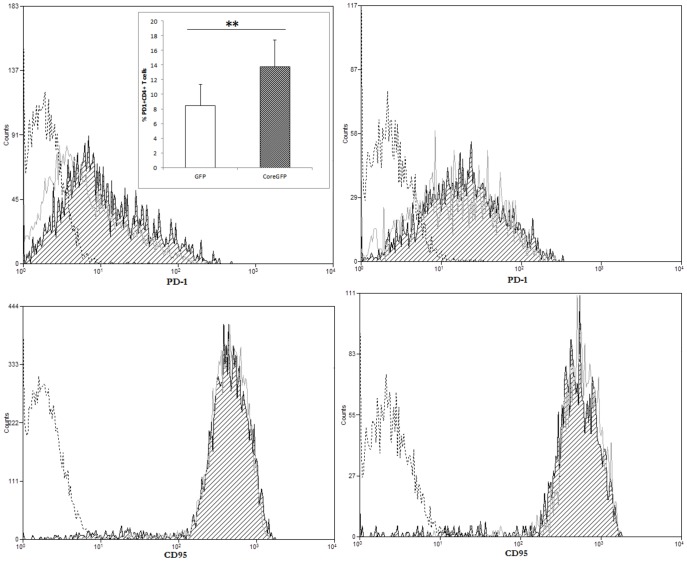
PD-1 expression increases in resting HCV-core expressing CD4+ T cells. Purified CD4+ T cells were transduced with lentiviral vectors coding for HCV-core or GFP as a control, rested, stimulated and stained for PD-1 or CD95 surface expression. Histograms represent PD-1 (upper panels) or CD95 (lower panels) expression after positive gating of transduced cells and exclusion of dead cells, in unstimulated (left panels) or stimulated (right panels) HCV-core (black striped curve) or GFP (grey open curve) expressing CD4+ T cells. A negative isotype control is represented in a dashed black open curve. A representative experiment out of four performed with different donors is shown. Bar diagram in insert corresponds to mean±SD of the median of fluorescence intensity (MFI) for GFP transduced (open bar) or HCV-core transduced (grey striped bar) CD4+ T cells (**p<0.01).

It has recently been shown that T-cells from chronic HCV patients, undergo increased apoptosis after TCR stimulation [69], thus we wanted to analyze whether expression of HCV-core was sufficient for the induction of PD-1 and/or CD95. As shown in figure 8, PD1 staining was increased in unstimulated resting HCV-core expressing CD4+ T cells compared to GFP expressing control cells with a shift in the median fluorescence intensity (MFI) (38.77% PD1+ cells in GFP expressing CD4+ versus 48.95% in HCV-core expressing CD4+ T cells) while differences disappeared upon stimulation. On the other hand, there was no change in CD95 expression in HCV-core transduced CD4+ T cells either resting or stimulated (Fig. 8).

### CD127 expression decreases, while TIM-3 expression increases in HCV-core expressing CD4+ T cells

**Figure 9:**
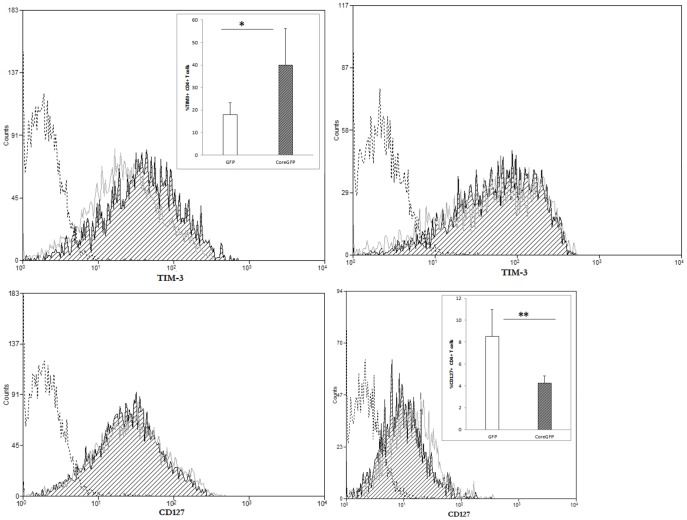
CD127 expression decreases, while TIM-3 expression increases in HCV-core expressing CD4+ T cells. Purified CD4+ T cells were transduced to express HCV-core or GFP as a control and stained for CD127 and TIM-3 surface expression. Histograms represent TIM-3 (upper panels) or CD127 (lower panels) expression after positive gating of transduced cells and exclusion of dead cells in unstimulated (left panels) or stimulated (right panels) HCV-core (black striped curve) or GFP (grey open curve) expressing CD4+ T cells. A negative isotype control is represented in a dashed black open curve. A representative experiment out of three performed with different donors is shown. Bar diagrams in inserts correspond to mean±SD of the median of MFI for GFP transduced (open bar) or HCV-core transduced (grey striped bar) CD4+ T cells (**p<0.01; *p<0.05).

Interestingly, high level of PD1 expression has been described in HCV infected patients and it has also been shown in exhausted T cells. Thus we analyzed the expression of receptors such as TIM-3 and CD127, which have been described in T_reg_ cells with an exhausted phenotype. MFI for TIM-3 staining was increased in unstimulated HCV-core CD4+ T cells with a correspondent increase in percentage (57.82 TIM-3+ cells in GFP expressing CD4+ T cells versus 67.34% in HCV-core expressing CD4+ T cells) (Fig. 9 left panel). As it occurred for PD-1, differences disappeared upon stimulation (Fig. 9 right panel). On the other hand, CD127 expression showed a decrease in HCV-core expressing CD4+ T cells but only after stimulation (57.71% CD127+ cells in GFP expressing CD4+ T cells versus 38.57% in HCV-core expressing CD4+ T cells) (Fig. 9 right panel), while there were no differences in the percentage of CD127 expression in unstimulated cells (Fig. 9 left panel).

### CCR7 expression decreases on resting HCV-core expressing CD4+ T cells

**Figure 10:**
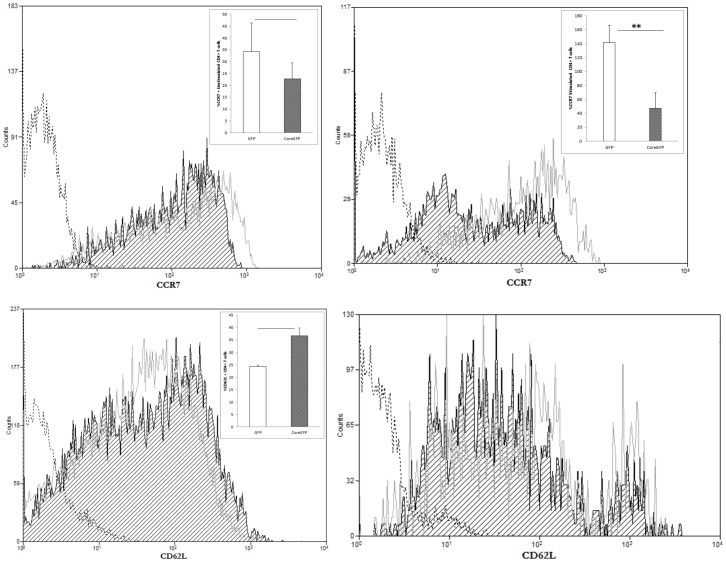
CCR7 expression decreases on resting HCV-core expressing CD4+ T cells. Purified CD4+ T cells were transduced to express HCV-core or GFP as a control and stained for CCR7 or CD62L surface expression. Histograms represent CCR7 (upper panels) or CD62L (lower panels) expression after positive gating of transduced cells and exclusion of dead cells in unstimulated (left panels) or stimulated (right panels) HCV-core (black striped curve) or GFP (grey open curve) expressing CD4+ T cells. A negative isotype control is represented in a dashed black open curve. A representative experiment out of three performed with different donors is shown. Bar diagrams in inserts correspond to mean±SD of the median of MFI for GFP transduced (open bar) or HCV-core transduced (grey striped bar) CD4+ T cells (**p<0.01).

**Figure 11:**
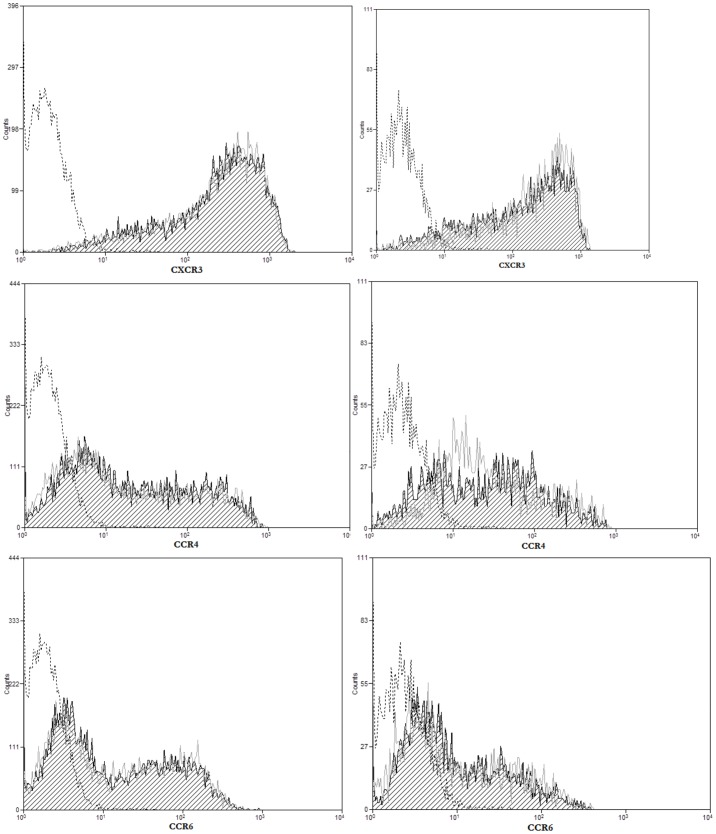
CXCR3, CCR4 and CCR6 expression do not change on HCV-core expressing CD4+ T cells. Purified CD4+ T cells were transduced to express HCV-core or GFP as a control and stained for CXCR3, CCR4 or CCR6 surface expression. Histograms represent CXCR3 (upper panels), CCR4 (medium panels) or CCR6 (lower panels) expression after positive gating of transduced cells and exclusion of dead cells in unstimulated (left panels) or stimulated (right panels) HCV-core (black striped curve) or GFP (grey open curve) expressing CD4+ T cells. A negative isotype control is represented in a dashed black open curve. A representative experiment out of three performed with different donors is shown.

We next wanted to analyze whether the effect observed in HCV-core expressing CD4+ T cells is related to a stop in effector cell differentiation, thus we analyzed markers of memory T cell such as CD62L or CCR7 (Fig. 9), as well as effector T cell type 1 (CXCR3) or type 17 (CCR4 and CCR6). As shown in figure 10, MFI for CCR7 staining slightly decreased in unstimulated HCV-core expressing CD4+ T cells with a correspondent change in percentage (87.6% CCR7+ cells in GFP CD4+ T cells vs. 81.94% in HCV-core CD4+ T cells), while upon stimulation it showed a significant decrease (88.52% GFP expressing CD4+T cells vs. 63.28% in HCV-core expressing CD4+ T cells) (Fig. 10). CD62L increases although not significantly in unstimulated HCV-core expressing CD4+ T cells (42.27% CD62L+ cells in HCV-core expressing CD4+ T cells versus 33.02% in GFP expressing CD4+ T cells) while differences decreased upon stimulation (Fig. 10). There were no differences in CXCR3, CCR4 and CCR6 expression between HCV-core transduced CD4+ T cells and GFP controls (Fig. 11).

## Discussion

It has long been observed that HCV chronically infected patients are not able to clear the infection, mainly because cellular immune responses are dysfunctional [70]–[72]. PBMC and particularly CD4+ T cells from chronically infected patients, show a decrease in IL-2 along with an increase in IL-10 secretion [73]–[75]. In recent years, the role of T_reg_ cells in the outcome of HCV infection is gaining much attention and an increase of several cell types with regulatory function have been described in HCV chronically infected patients [22]. In this regard, most studies have focused on CD4+CD25+ T cells, describing an increase in the frequency of peripheral CD4+CD25+ [17], [22] that are able to suppress HCV-specific and interestingly, non-specific CD8+ T cell responses [20], [27]. IL-10 producing Tr1 cells can also be isolated and cloned from patients with chronic HCV infection, but not from patients who cleared the infection [17], [76]. Some other cell types with regulatory functions, such as TGF-β-producing CD4+ T cells [12], [17] and regulatory CD8+ T cells [77] have also been described in chronic HCV infection. Overall, these data suggest an important role for cells with regulatory activity in the course and outcome of HCV infection.

There are several reports showing an increase in CD4+CD25+ T_reg_ cell frequency in liver infiltrates displaying also an exhausted phenotype [24], [25], [78]–[80], as well as in peripheral blood of chronically infected patients when compared to those that spontaneously recovered or to healthy controls [17], [23]. It has been shown that T_reg_ cells from chronically infected subjects not only suppress proliferation and function of HCV-specific CD4+ and CD8+ T cells, but also T cell responses against other viruses such as influenza or EBV, suggesting a possible non-specific pattern of suppression [20], [27].

In this work, we show that expression of HCV-core is able to induce de novo generation of CD4^+^CD25^+^CTLA4^+^Foxp3^+^CD127^low^ T cells with a modest regulatory function and partial exhaustion characteristics, suggesting that the increase in T_reg_ cells found in peripheral blood from chronically infected patients may be developed at least partially in the periphery upon internalization of viral particles or proteins. Our results are in keeping with results were addition of HCVne to peripheral T cells induced expression of CTLA-4 and CD25, while CD127 expression showed a gradual decrease compatible with the regulatory phenotype with exhausted features [40]. Although the effect of exogenously added HCV-core has been shown to be partially mediated by interaction with the complement receptor gC1qR [39], considering that interaction with the gC1qR do not replicate all HCV-core effects [81], gC1qR is displayed not only in CD4+ but also in CD8+ T lymphocytes [81] while we only see a direct effect on CD4+ and HCVne particles are shown to be internalized [40], [59], some effects induced by HCVne could be performed by the presence of HCV-core inside T cells, ensuing in a scenario similar to our experimental setting. Thus, our findings add a new possibility on the generation of T_reg_ cells in the periphery induced by intracellular expression of a single viral protein. The phenotype described in HCV-core expressing CD4+ T cells aids in the explanation of several clinical and experimental results as it resembles clinical observations recorded for chronically infected patients, where specific CD4+ T cells show an anergic phenotype characterized by a decrease in IL-2 and IFN-γ production, high IL-10 production and decreased proliferation [12]. And it is also in agreement with results showing that immunization with HCV-core but not with NS3, led to a poor proliferative response, reduced induction of cytokines such as IFN-γ and IL-2, development of CD4^+^CD25^+^CTLA4^+^Foxp3^+^CD127^low^ T cells with regulatory functions, exhaustion characteristics and increased production of IL-10 [23], [82]. Our initial results from unfractionated PBMC, showed a decrease in IL-2 production by both CD4+ and CD8+ T cells transduced with HCV-core, while expression of HCV-core on purified CD4+ or CD8+ T cells had an inhibitory effect only on CD4+ while CD8+ T cells showed a percentage of IL-2 secreting cells similar to controls. These results indicate that HCV-core transduction has a direct effect on CD4+ cells while the effect on CD8+ cells is mediated by CD4, in agreement with what has been reported for HCVne uptake by PBMCs [40] that has been further confirmed in the present work by co-culture experiments.

Our results regarding the regulatory effect exerted by HCV-core expressing CD4+ T cells on CD8+ T cells are in agreement with several results showing a decrease in CD8+ T cell function in HCV chronically infected patients [22], [83]–[88], many of them invoking CD4+ T cell regulation and/or a decrease in CD4+ T cell help [14], [89]. Our results on CD8 regulation are also relevant *in vivo* in the context of HCV induced liver pathophysiology were CD4+ Foxp3+ T cell have been shown to be predominantly localized in piecemeal and lobular necrosis, in contact with CD8+ T cells [90]. Thus, Treg cells within HCV infected livers have direct access to CD8+ T cells *in vivo*.

Although, in the context of HCV liver fibrosis a total increase in CD8+ T cells number [91] or a relative increase compared to CD4+ T cells [92] have been reported, other authors showed that differences in the periphery were not significant being mainly confined to the intrahepatic lymphocyte composition with negative detection in normal livers [92].

Li et al. have shown that CD4+CD25+Foxp3+ T cells are increased upon addition of HCV-core derived peptides to PBMC cultures from healthy donors or HCV chronically infected patients [93]. These results were interpreted as priming, induction or expansion of HCV-core specific T_reg_ cells. In our hands, Jurkat cells [21] and CD4+ T cells from healthy donors became Foxp3+ as well as suppressive, due to intracellular HCV-core expression. Thus our results add a new possibility as CD4+ cells from chronically infected patients may express HCV-core, either due to infection [43]–[45], [55], [94], [95] or uptake of the viral protein [40], [53], [57] shown to be present in the serum [56] and CD4+ T cells expressing HCV-core can be transformed into T_reg_ cells. Thus presence of intracellular viral proteins in CD4+ cells could play a role in the generation of T_reg_ cells observed in HCV chronically infected patients.

The effect of HCV-core in cytokine expression has already been reported by us and others in tumor cell lines, thus HCV-core transduced CD4+ Jurkat cells show an “anergic-like” phenotype characterized by a decrease in IL-2 production upon stimulation, increase in IL-10 production [21], [37] and upregulation of anergy associated genes [36] as previously described in mouse [96]. In the present work, we show that in primary human CD4+ T cells, HCV-core expression induced a decrease in IL-2 and IFN-γ production and interestingly, HCV-core transduced CD4+ T cells had elevated IL-10 levels compared to control cells, both in basal conditions and after CD3 plus CD28 stimulation, being a percentage of CD4+Foxp3+ T cells, the IL-10 secreting cells. On the contrary, TGF-beta levels in HCV-core expressing CD4+ cells were similar to controls.

In the present study, the cytokine expression pattern shown by HCV-core expressing CD4+ T cells resembles that observed in CD4+ T cells from HCV-chronically infected patients, who show a decrease in IFN-γ and IL-2 production and high IL-10 secretion [12], [97]–[99]. There are some exceptions to the IFN-γ decrease [100], while in some other series IFN-γ reduction was only apparent upon stimulation [74], consistent with our results. A decrease in IFN-γ production has also been shown upon infection of primary CD4+ cells with HCV [51].

Elevated IL-10 basal levels have also been detected in PBMC from chronically infected patients [73] and in HCV-core expressing Jurkat cells [21], [37] and in terms of cytokine production and immune suppression, our results are also in line with the effects induced by HCV-core expression in a transgenic mouse model [101].

In our experiments, HCV-core transduced CD4+ T cells showed an increased expression of CTLA-4 and Foxp3. CTLA-4 inhibits T cell responses and has been described to be induced in some T_reg_ cell populations [102]–[105] and to be implicated in anergy induction *in vivo* [102] and Foxp3 has been shown to be sufficient for the suppressor activity of CD4+CD25+ T cells: as it has been reported that expression of Foxp3 in CD4+CD25-Foxp3- T cells confers a regulatory function on those cells [61]–[63].

Overall, our data show that HCV-core protein induces a suppressor phenotype in CD4+ T cells. HCV-core expressing CD4+ T cells showed an anergic phenotype, being unresponsive to TCR stimulation and being able to suppress polyclonal CD4+ and CD8+ T cell activation. Moreover, HCV-core expressing CD4+ T cells displayed an increased expression of molecules directly implicated in the inhibition of T cell activation, such as CTLA-4 and IL-10, as well as Foxp3, the most accepted marker for CD4+CD25+ T_reg_ cells.

Here we also show that HCV-core expressing CD4+ T cells upregulate PD-1 and TIM-3 and downregulate CD127.

These findings are in agreement with reports in patients with HCV chronic infection [23], [80], [106], [107], where PD-1 upregulation on peripheral CD4+CD127^low^CD25^hi^ Foxp3^+^ T_reg_ cells has been shown, with CD4+ T cell responses, including virus specific IFN-γ production, being severely suppressed through the PD-1/PD-L1 pathway [108], and Franceschini et al. have reported a high expression of PD-1 on T_reg_ cells, particularly those infiltrating the liver with the frequency of PD-1+ T_reg_ cells being greater than the frequency of PD-1+ CD4+ effector T cells [80], [109].

It has been reported that T_reg_ cell suppressive function is regulated by PD-1/PD-L1 pathway in HCV patients [80], [106]. Moreover PD-1/PD-L1 pathway has been critically involved in HCV long-term persistence and it has even been regarded as a potential novel target for restoring function of exhausted HCV-specific T cell responses [110], [111].

Overexpression of a single viral protein is sufficient to induce some of the features described for HCV infected patients. Interestingly, PD-1 is up-regulated through NFAT activation [112] and we and others have shown that HCV-core expression is sufficient to activate NFAT [36], [113].

An increased expression of TIM-3 has also been reported as an additional molecule that mediates T cell exhaustion in chronic HCV infection [114]. A significantly higher percentage of total CD4+ and CD8+ T cells and HCV-specific CTLs within the hepatic compartment co-express TIM-3 and PD-1 [114], in agreement with our results and with the hypothesis stating that the liver is enriched in T cells that are functionally exhausted. Moorman et al. found TIM-3 upregulated, not only on IL-2–producing CD4^+^CD25^+^Foxp3^−^ effector T cells, but also on CD4^+^CD25^+^Foxp3^+^ T_reg_ cells, which accumulate in the peripheral blood of chronically HCV-infected individuals [115].

Additionally, Ji et al. have recently shown that TIM-3/Gal-9 pathway not only regulate negatively effector T cells but also regulate HCV-mediated T_reg_ cell development, showing that HCV-infected human hepatocytes upregulate TIM-3 expression in co-cultured human CD4+ T cells, driving conventional CD4+ T cells into CD25+ Foxp3+ T_reg_ cells [116].

The downregulation of CD127 shown in our work is in agreement with reports indicating that CD127 expression decreases in CD4^+^CD25^+^Foxp3^+^ T cells during chronic HCV infection [23], [79], [108], acute HCV infection associated with viral persistence on both CD4+ and CD8+ T cells irrespective of naïve/memory phenotype [117] and after HCV-core stimulation [23]. Although Doumba et al. showed elevated CD127 expression in peripheral T cells 12 h after incubation with HCVne, they showed a subsequent decrease [40] also in agreement with our results. Thus, given the fact that IL-7 supports the emergence and survival of memory CD4+ and CD8+ T lymphocytes, the differential CD127 expression in the early stages of infection can be partly responsible for viral persistence [117].

Interestingly, in this study, we also found that HCV-core protein induces variations in the expression of receptors involved in chemotactic functions, such as CCR7 and CD62L in CD4+ T cells. Specifically, HCV-core protein induces in CD4+ T cells a decreased expression of CCR7 and increased expression of CD62L. Although few studies analyze the expression of CCR7 and CD62L in T cells during HCV infection and most of them focus on CD8+ T cells [24], [25], [117], Quiroga et al. also showed CD62L expression on virus-specific CD4+ T cells [118]. While the role of CD62L on effector T cells has been extensively investigated, its function on T_reg_ cells is largely unexplored. Interestingly, during clinical remissions of relapsing remitting multiple sclerosis, it has been described that T_reg_ cells expressing CD62L^high^, accumulate at the site of autoimmune inflammation. CD62L^high^ cells express large quantities of CTLA-4, ICOS and TGF-β and are more potent in suppressing proliferation in effector T cells than CD62L^low^ T_reg_ cells [119], [120].

Regarding CCR7 downregulation, it has been shown that recirculation of naive-like T_reg_ cells through lymph nodes is dependent on CCR7, while CCR7 deficiency in effector/memory-like T_reg_ cells promotes their accumulation in inflamed sites [121], thus HCV-core expressing CD4+ T cells will preferentially home into inflamed liver where it could downregulate anti HCV immune responses. Accordingly Claassen et al. have described an extensive lymphocyte infiltration containing abundant numbers of CD4+Foxp3+ T_reg_ cells in HCV-infected livers, while was absent from healthy liver. Interestingly, these T_reg_ cells were more abundant in those HCV-infected livers showing only limited fibrosis [24]. Relevant to these findings, the frequencies of intrahepatic Foxp3+ T_reg_ cells correlated directly with plasma HCV viral load and inversely with histological injury, indicating that these liver-infiltrating T_reg_ cells were actively modulating the overall effector response to HCV infection in the liver [80], [109].

We did not find any significant difference in other chemokine receptors such as CXCR3 and CCR6 in agreement with results by Kared et al. [107].

We did not find either significant differences in the expression of CD95. Although it has been shown that T lymphocytes from chronic HCV infected patients are primed for activation-induced apoptosis [69] we did not previously find an increase in apoptosis by expression of HCV-core alone [36], which is in agreement with the absence of Fas upregulation found here. Panasiuk et al., found an upregulation of Fas in CD4+ T cells apparently associated with inflammatory activity and fibrosis in the liver and only in advanced stages of liver disease whereas our experimental system explore alterations early after expression of HCV-core in purified CD4+ T cells.

Overall, our data show that HCV-core protein induces a suppressor phenotype in CD4+ T cells. HCV-core expressing CD4+ T cells show an anergic phenotype, being unresponsive to TCR stimulation and being able to suppress CD4+ and CD8+ T cell activation. Moreover, HCV-core expressing CD4+ T cells displayed an increased expression of molecules implicated in the inhibition of T cell activation, such as CTLA-4, IL-10 as well as Foxp3, the most accepted marker for CD4+CD25+ T cells; and upregulation of molecules involved in mechanism of exhaustion in T cells, such as PD-1, TIM-3 and CD127.

Although the fine classification of CD4+ T_reg_ cells remains controversial, the CD4+ T cells expressing HCV-core shown in our work meet their major hallmark: suppressive ability and Foxp3 expression [122]. Taken together the additional expression pattern and the cytokine secretion profile found in HCV-core expressing CD4+ T cells in this study, we can define these cells as T_reg_ cells with exhausted T cell features.

In HCV infected patients, T_reg_ cells contribute critically to suppression of HCV-specific lymphocyte proliferation, differentiation, cytokine secretion and subsequently to the pathology progression [123]. HCV patients have a higher number of T_reg_ cells in peripheral blood than healthy individuals [18], [24], [124] and depletion of CD4+CD25+T cells enhances antigen-specific CD4+ and CD8+ T cell proliferation and response [16]-[18],[20]. Our results give an example for the generation of cells with regulatory activity by the expression of a single viral protein.
